# Acute ischemic stroke in young women secondary to atherosclerosis: a unique ophthalmic presentation with central retinal artery occlusion: a case report

**DOI:** 10.11604/pamj.2022.41.189.33147

**Published:** 2022-03-09

**Authors:** Salma Moutamani, Taha Boutaj, Amine Ennejjar, Wiame Touil, Abdellah Amazouzi, Ouafa Cherkaoui

**Affiliations:** 1 Ophthalmology Department “A”, Ibn Sina University Hospital (*Hôpital des Spécialités*), Mohammed V University, Rabat, Morocco,; 2 Neurology Department “B”, Ibn Sina University Hospital (*Hôpital des Spécialités*), Mohammed V University, Rabat, Morocco

**Keywords:** Atherosclerosis, ischemic stroke, neuro-ophthalmology, retina, case report

## Abstract

Central retinal artery occlusion (CRAO) is a rare condition. It is a diagnostic and therapeutic emergency. CRAO is analogous to an acute stroke of the eye. The disease usually affects patients after 60 years of age, and rarely young persons. The risk factors for a CRAO are similar to ischemic stroke. It is due to embolic, thrombotic, or coagulation disorders. Typically, patients with acute CRAO present monocular, painless, and severe loss of vision. We report a rare case of a young 33-year-old woman who presented an atypical ischemic stroke revealed by a unilateral CRAO. This case reports a rare condition affecting a young patient, and highlights the interest of ophthalmologic examination in the diagnostic of a neuro disease and the early management of ischemic stroke.

## Introduction

Central retinal artery occlusion (CRAO) is a rare condition, affecting approximately one of 10,000 people, and two men for every woman, after the age of 60, rarely young people [1]. It is due to a thrombotic or embolic obstruction of the central retinal artery, or to coagulation disorders [1,2] requiring careful etiological investigation [2]. Central retinal artery occlusion (CRAO) is a diagnostic and therapeutic emergency because it is associated with general pathologies which can be fatal [3].

## Patient and observation

**Patient information:** we report the case of a 33-year-old woman, with no particular antecedents, who arrived at the ophthalmology emergency presenting sudden painless loss of vision of the left eye in the morning.

**Figure 1:**
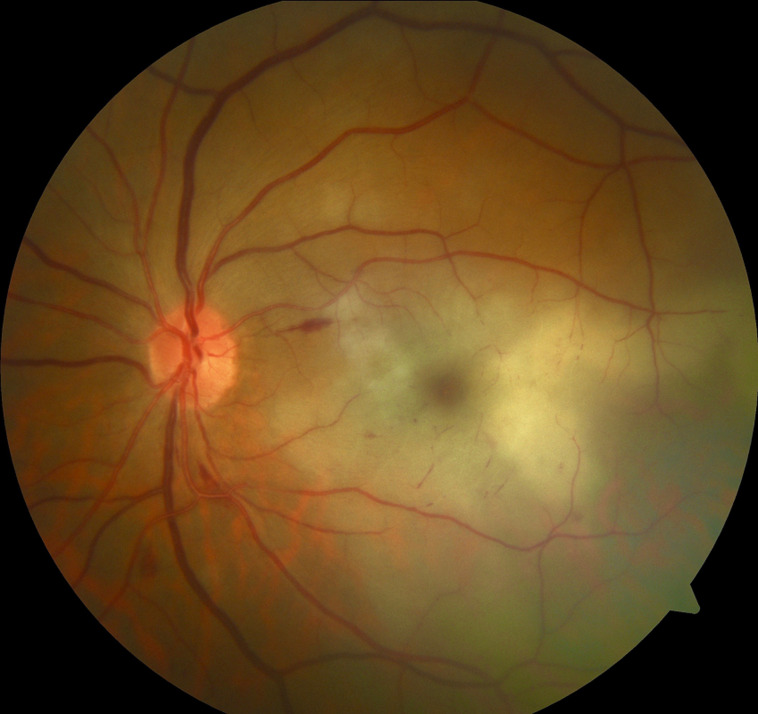
fundus-copy of the left eye showing white retinal edema of the posterior pole with a discreet red reflection of the macula

**Clinical findings:** general examination found a high Body Mass Index (BMI) at 35 kg/m^2^. On the ophthalmologic examination of the left eye, visual acuity was reduced to minimal light perception. The pupillary light reflex was absent. Intraocular pressure was normal. Slit-lamp biomicroscopic examination found a good anterior segment with filamentous vitreous. Funduscopy of the left eye objectives white retinal edema of the posterior pole with a reddish reflection of the macula (Figure 1). The right eye´s examination was unremarkable.

**Figure 2:**
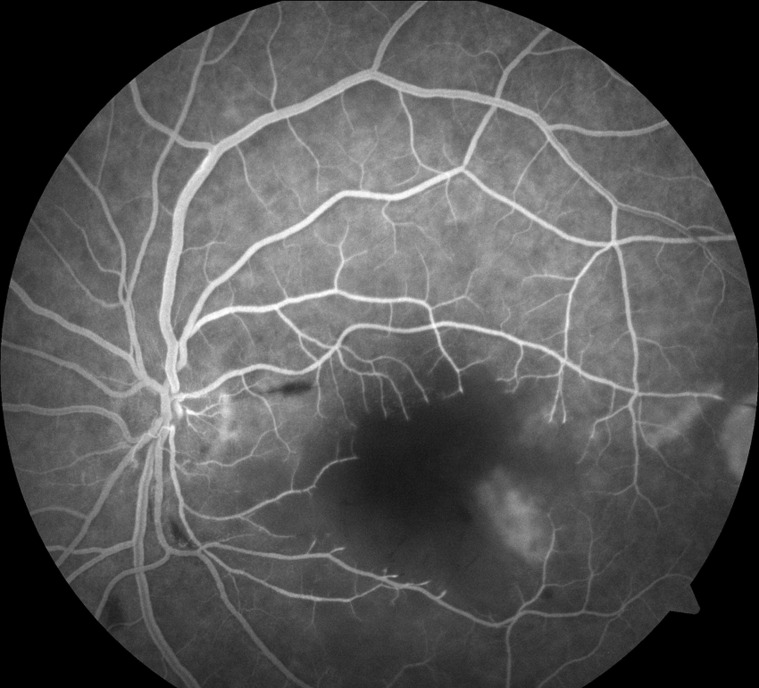
fluorescein angiography showing absence of flow in the peri-macular arterioles and enlargement of the central avascular zone related to ischemic edema in the left eye

**Figure 3:**
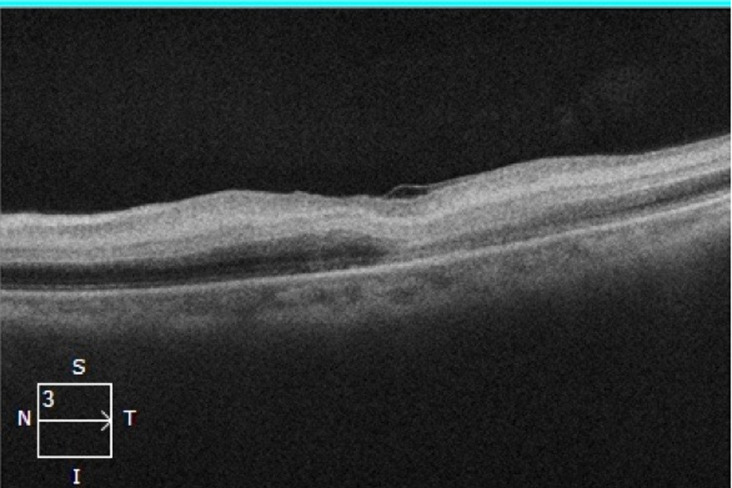
macular optic coherence tomography of the left eye confirming ischemic edema with hyper-reflectivity predominant on the inner layers of the retina

**Figure 4:**
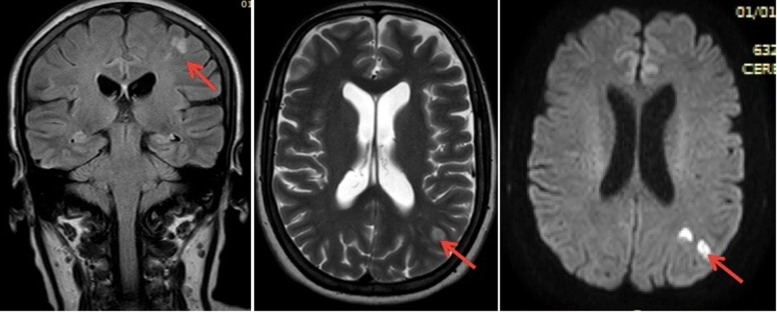
cerebral MRI revealing an acute ischemic stroke constituted on the left: cortico-subcortical area in the hyper signal

**Figure 5:**
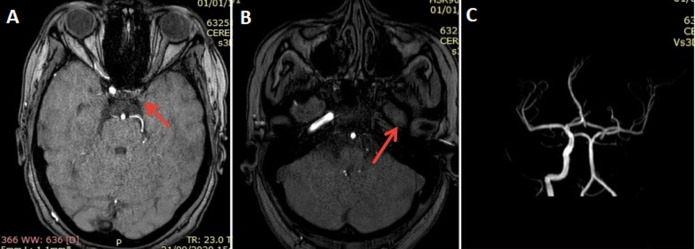
cerebral MRI: 3D TOF MRA sequence: A) absence of flow at the level of the left internal carotid artery at the level of its intra-cavernous; B) intra petrous portion, 3D reconstruction; C) absence of flow at the level of the left common carotid artery (ACC) and internal carotid artery (ACI)

**Figure 6:**
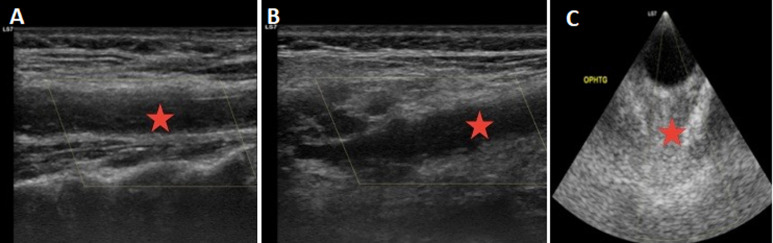
echo-doppler of the supra-aortic vessels (A, B): endoluminal material at the level of the left common carotid artery extended to the carotid bulb with no color doppler plug; C) absence of color doppler plug in the left ophthalmic artery

**Diagnostic assessment**: fluorescein angiography revealed a slow and progressive filling of the arteries with interruption of the flow at the level of the peri-macular arterioles and widening of the central avascular zone (Figure 2). Macular Optic Coherence Tomography (macular OCT) revealed ischemic edema in the inner layers of the retina (Figure 3). The diagnosis of CRAO was retained. Because of neurological symptoms, a magnetic resonance angiography was realized revealing an acute ischemic stroke on the junctional territories of the anterior, posterior, and left middle cerebral artery, and occlusion of the common carotid artery, the left internal and external carotid artery (Figure 4, Figure 5). Echo-doppler of the supra-aortic vessels showed thrombosis of the left common carotid artery extending to the ipsilateral internal carotid artery (Figure 6). A cardiology consultation ruled out all causes of cardiac origin. A biological assessment including lipid profile, glycemic balance, and a Complete Blood Count (CBC) detected hypercholesterolemia and unbalanced diabetes.

**Therapeutic intervention:** the patient was hospitalized in the neuro-ophthalmology department for initiation of the treatment and the management of her cardiovascular risk factors.

**Follow-up and outcomes:** after two weeks of hospitalization, visual acuity improved to 1/10. Follow-up after 1 month was uneventful.

## Discussion

First described by von Graefes in 1859 [4], CRAO is analogous to an acute stroke of the eye and is an ophthalmic emergency. The same atherosclerotic risk factors that predispose to cerebrovascular and cardio disease are present in CRAO [1]. It affects old people after 60 years old. It rarely affects young ones, mainly in systemic diseases. Patients presenting with CRAO often have a previously undiagnosed vascular risk factor that may be amenable to medical or surgical treatment [5]. About the clinical presentation, patients with acute CRAO present with painless, monocular, and severe loss of vision. Visual acuity following a CRAO varies greatly, ranging from near-normal visual acuity in the presence of a cilioretinal artery sparing the fovea, to counting fingers or worse vision in most patients [6]. CRAO has a poor spontaneous recovery rate. It depends on many factors, including the presence of a patent cilioretinal artery.

Treatment of CRAO aims acute reperfusion of the central retinal artery, prevention of ocular complications, and vascular review to prevent ischemic diseases. Currently, conventional therapy consists of dislodging emboli, reducing intraocular pressure and increasing retinal blood flow, improving retinal circulation, vasodilating the ocular blood supply, decreasing retinal edema, maintaining retinal oxygenation until spontaneous reperfusion, and acting on the thrombus [7]. None of these treatments have proven effective and their use is based on some reports and small case series.

## Conclusion

Patients presenting with features of CRAO require a thorough systemic evaluation. It is a diagnostic and therapeutic emergency. There is a high association between CRAO and vascular stroke. Treatment of CRAO must aim reperfusion of the central retinal artery, and prevention of vascular complications.
